# Roles of *Streptococcus mutans*-*Candida albicans* interaction in early childhood caries: a literature review

**DOI:** 10.3389/fcimb.2023.1151532

**Published:** 2023-05-16

**Authors:** Yifei Lu, Yifan Lin, Mingyun Li, Jinzhi He

**Affiliations:** ^1^State Key Laboratory of Oral Diseases and National Clinical Research Center for Oral Diseases, Sichuan University, Chengdu, China; ^2^Department of Cariology and Endodontics, West China Hospital of Stomatology, Sichuan University, Chengdu, China

**Keywords:** early-childhood caries, *Candida albicans*, *Streptococcus mutans*, interkingdom interaction, symbiosis, antagonism

## Abstract

As one of the most common oral diseases in kids, early childhood caries affects the health of children throughout the world. Clinical investigations show the copresence of *Candida albicans* and *Streptococcus mutans* in ECC lesions, and mechanistic studies reveal co-existence of *C. albicans* and *S. mutans* affects both of their cariogenicity. Clearly a comprehensive understanding of the interkingdom interaction between these two microorganisms has important implications for ECC treatment and prevention. To this end, this review summarizes advances in our understanding of the virulence of both *C. albicans* and *S. mutans*. More importantly, the synergistic and antagonistic interactions between these two microbes are discussed.

## 1 Introduction

Early childhood caries is defined as “the presence of one or more decayed (non-cavitated or cavitated lesions), missing (due to caries) or filled tooth surfaces in any primary tooth” in a child under the age of six (American Academy of Pediatric Dentistry, 2021). Although significant progress against ECC has been made in pediatric dentistry, it still affects kids throughout the world. According to 72 global studies which measured the prevalence of ECC in preschoolers between 1998 and 2018, the average prevalence of ECC in kids at 1-year-old was 17%, while the prevalence of ECC in toddlers at 2-year-old sharply rose to 36% (Tinanoff et al., 2019). Worse still, the prevalence of ECC increases with the development of children. Specifically, the prevalence of ECC in 3, 4 and 5-year-old preschoolers was 43%, 55% and 63%, respectively (Tinanoff et al., 2019). ECC impacts children and their parents or caregivers in different ways. Apart from dental pain and abscess, children with ECC suffer a higher risk of hospitalization and emergency room visits, treatment costs, school time loss, poor learning ability and life quality, low growth parameters (weight, height), sleep disorders, self-esteem setbacks and negative social interactions (Sachdev et al., 2016; Vieira-Andrade et al., 2016; Xiao et al., 2018a; Pierce et al., 2019).

ECC is a multifactorial disease. The prerequisites of ECC include the synchronized appearance of susceptible hosts, cariogenic microbes, and cariogenic substrate from food present for a sufficient length of time. Microbes are the initial factor of ECC. For quite a long time, oral microbiologists focused on the role of oral bacteria in the pathogenesis of ECC. *Streptococcus mutans*, a gram-positive facultative anaerobe, has long been taken as the main etiological factor of ECC. It is important to keep in mind that, the oral cavity is colonized by microbes from different domains, including eukaryotic cells, prokaryotic cells, archaea, and viruses. Although bacteria-targeting studies provide important clues to understand how microbes destroy the hard tissues of mammalian teeth, the roles of other microorganisms should not be ignored. In recent years, *Candida albicans*, a eukaryote and an opportunistic fungus, has been frequently detected in children with ECC. Studies revealed that the detection of *C. albicans* is positively related to the severity and incidence of ECC (Cui et al., 2021), suggesting its role in the occurrence of ECC. Importantly however, within the oral cavity, *S. mutans* and *C. albicans* actively interact with each other, and their inter-kingdom interactions mediate both of their cariogenicity (Falsetta et al., 2014; Kim et al., 2017; Xiao et al., 2018b; Sridhar et al., 2020). In this review, the virulence of *S. mutans* and *C. albicans* is present first, and then their complicated interactions as well as the influence of these interspecies interactions on the occurrence of ECC are discussed.

## 2 Streptococcus mutans

*S. mutans* is naturally present in the human oral cavity. To survive in the harsh environment of the human mouth, *S. mutans*, as well as other oral microbes, forms a highly organized microbial community termed as biofilm (Marsh and Zaura, 2017). Biofilm is a complex structure composed of aggregated microbial cells and microbially produced extracellular polymeric substances (EPS) (Marsh and Zaura, 2017). The construction of biofilm begins when saliva components selectively adsorb to the tooth surfaces, forming a thin acellular homogeneous organic membrane termed as acquired enamel pellicle. Acquired enamel pellicle provides binding sites for oral microorganisms including *S. mutan*s. The surface proteins P1 (also known as PAc, SpaP, Ag I/II protein) of *S. mutan*s can selectively bind to salivary lectins in acquired enamel pellicle, and this binding process enable the initial colonization of *S. m*utans. After initial binding, microbes including *S. mutans* start to proliferate and produce EPS, forming a stable three-dimensional community that contains channels to effectively distribute nutrients, oxygen and signaling molecules. *S. mutans* significantly promotes biofilm formation through sucrose-dependent and -independent ways. The sucrose-dependent mechanism mainly relies on extracellular glucose transferase secreted by itself. There are three extracellular glucose transferases secreted by *S. mutans*, including GtfB, GtfC, and GtfD. GtfB mostly produces viscous water-insoluble polysaccharides from sucrose, while GtfC synthesizes a mixture of insoluble and soluble polysaccharides from sucrose. GtfD predominantly catalyzes sucrose to be soluble polysaccharides (Bowen and Koo, 2011; Krzyściak et al., 2014). Water-insoluble polysaccharides are the major component of EPS. Water-insoluble polysaccharides work as glue to facilitate bacterial adherence and accumulation. They also constitute the protective barrier for residing microbes, and provide the biofilm with mechanical stability (Cugini et al., 2019).

*S. mutans* promote the occurrence of dental caries including ECC by generating organic acids through carbohydrate metabolization. Carbohydrates including sugars from the environment are transported into *S. mutans* cells mainly through the phosphoenolpyruvate dependent phosphotransferase system (PEP-PTS). This system catalyzes the transportation and phosphorylation of monosaccharides, disaccharides, amino sugars, polyols, and other sugar derivatives. The bacteria ferment the phosphorylated sugar into pyruvate by glycolysis. Pyruvate then is catalyzed to be organic acids such as lactic acid and formic acid through a series of branched chain pathways. These organic acids lower the pH of the local microenvironment, which is the direct cause of tooth surface demineralization. Majority of sucrose (> 95%) is internalized through the PEP-PTS system, and the rest is extracellularly metabolized by Gtfs and fructose transferases (Ftfs) (Vadeboncoeur and Pelletier, 1997; Lemos et al., 2019). Other two binding protein-dependent carbohydrate transport systems, the multiple-sugar metabolism system (Msm) and maltose/maltodextrin ABC transporter, are also involved in the sucrose uptake of *S. mutans* (Zeng and Burne, 2013).

Acid resistance is a significant survival advantage and virulence factor of *S. mutans*. *S. mutans* utilizes a series of adaptation mechanisms to respond to the acid-damage. One of these mechanisms is called acid tolerance response. ATR helps *S. mutans* maintain the cytoplasm at a neutral level compared to extracellular space when the environment becomes acidic (Baker et al., 2017). First, the F1F0-ATPase system within the *S. mutans* cells serves as the proton pump and the primary mechanism to maintain pH homeostasis. In an acidic condition, the F1F0-ATPase system is activated, consequently, protons are pumped out of the cell (Bender et al., 1986). Second, in response to the acidification of its environment, *S. mutans* increases the proportion of monounsaturated membrane fatty acids. The monounsaturated membrane fatty acids decrease the permeability of extracellular protons (Fozo and Quivey, 2004; Matsui and Cvitkovitch, 2010). *S. mutans* also secretes cardiolipin (Macgilvray et al., 2012), an important acid resistant substance. In terms of alkali production to cope with acid stress, *S. mutans* is urease and ADS negative, both of which are utilized by oral streptococci to synthesize neutralizing molecules urea and/or ammonia. Instead, *S. mutans* has an agmatine deiminase system (AgDS) encoded by the agmatine-inducible *aguBDAC* operon. The AgDS catalyzes ammonia, CO_2_, and ATP production (Chakraborty and Burne, 2017). Although AgDS does not seem to have a significant effect on environmental alkalization, the ammonia produced internally may contribute to the neutralization of cytoplasmic pH. In addition, Malolactic fermentation (MLF) could be also helpful for the alkalization of cytoplasm. MLF converts malate to less acidic lactate and CO_2_ (Chakraborty and Burne, 2017). Interestingly, this fermentation process can also lead to ATP synthesis through the reversible action of the F1−F0-ATPase (Chakraborty and Burne, 2017). In addition, when growing at low pH, the branched-chain amino acid (BCAA) biosynthesis of *S. mutans* was up-regulated. Pyruvate, the key metabolic intermediate, can be redirected to BCAA biosynthesis, therefore reducing acid end products, and maintaining the intracellular pH to alleviate acid stress (Len et al., 2004). Meanwhile, amino acids biosynthetic genes such as *ilvC* and *ilvE* related to the biosynthesis/degradation of branched-chain amino acids and the production of branched-chain fatty acids were identified as being up-regulated (Santiago et al., 2012). Last but not the least, *S. mutans* encodes DNA/protein repairing enzymes, proteases and chaperones that can fix protein and DNA damages in an acidic environment.

## 3 Candida albicans

*Candida* is the most detected fungal species in the oral cavity, especially *C. albicans* (Witherden et al., 2017; Delaney et al., 2019). It is a polymorphic organism that grows in yeast or filamentous fungal filament or pseudo-hyphae. The ability to switch between yeast and filamentous forms is critical for its virulence. External environmental signals and internal regulation are involved in the regulation of yeast filamentous transformation of *C. albicans*. External signals include temperature, pH, CO_2_ concentration, serum, and malnutrition. Acidic pH, low temperature and rich nutritional conditions are conducive to *C. albicans* into yeast form (Lopes and Lionakis, 2022). Internal regulation includes signaling pathways and the phenotype conversion system called as white -opaque transition (Huang, 2012; Tao et al., 2022). *C. albicans* can adhere to the tooth enamel, and initial adhesion occurs through a strong interaction between yeast cell wall-associated adhesins and the salivary pellicle (Gunaratnam et al., 2021). Children with ECC had higher rates of *C. albicans* in saliva, dental plaque and infected dentin samples compared to kids without carious lesions (Moalic et al., 2001). Children with oral *C. albicans* have a higher risk for ECC (> 5 times) than children without *C. albicans* (Xiao et al., 2018b). The detection frequency of *C. albicans* in ECC was higher than that in caries cases not belonging to ECC and caries-free groups (Carvalho et al., 2006). The total loads of *C. albicans and S. mutans* in the supragingival dental plaque of children with ECC increase with the percentage of active carious lesions and the severity of dental caries (Sridhar et al., 2020). These studies, combined with others suggested there is a strong correlation between *C. albicans* and ECC.

It has been proved that *C. albicans* can produce acid (Klinke et al., 2011). *C. albicans* metabolized carbohydrates such as glucose from food, which causes the reduction of the pH in the growing environment from pH 7 to 4 (Fakhruddin et al., 2021). When the pH is below 5.5, the acidification of *S. mutans* decreased greatly and stopped at pH 4.2 (De Soet et al., 1991). However, *C. albicans* still maintains its acid production ability even at pH 4.0 (Klinke et al., 2009). The main organic acid synthesized by *C. albicans* were pyruvate and acetate (Klinke et al., 2009). *C. albicans* has a greater ability to dissolve hydroxyapatite, which is about 20 times higher than that of *S. mutans* (Nikawa et al., 2003).

*C. albicans* has a high affinity to acquired enamel pellicle. Scanning electron microscopy showed that *C. albicans* initially combined with the *in-situ* pellicles on enamel, indicating that yeast cells attached to the enamel surface were in close contact with the salivary pellicle (Rocha et al., 2018; Gunaratnam et al., 2021). *C. albicans* adheres to hydroxyapatite through electrostatic interaction (Nikawa et al., 2003). In recent years, two different patterns of *Candida* colonization have been found. One is the establishment of a mycelial network with bacteria, and the other is forming a spatial arrangement with *Streptococcus* (Dige and Nyvad, 2019).

Secretory aspartyl proteases (Sap) is a vital virulence factor of *C. albicans*. Sap1 may play important roles in the development of severe early childhood caries (S-ECC) (Li et al., 2014). *C. albicans* is the main producer of hydrolase, secreting enzymes such as protease, hemolysin, phospholipase, collagenase and so on, et al. *C. albicans* also has significantly higher protease activity and phospholipase activity. The study also showed that the phospholipase activity of isolated *C. albicans* was may be positively correlated with protease activity.

## 4 Interactions between *S. mutans* and *C. albicans*

**Figure 1:**
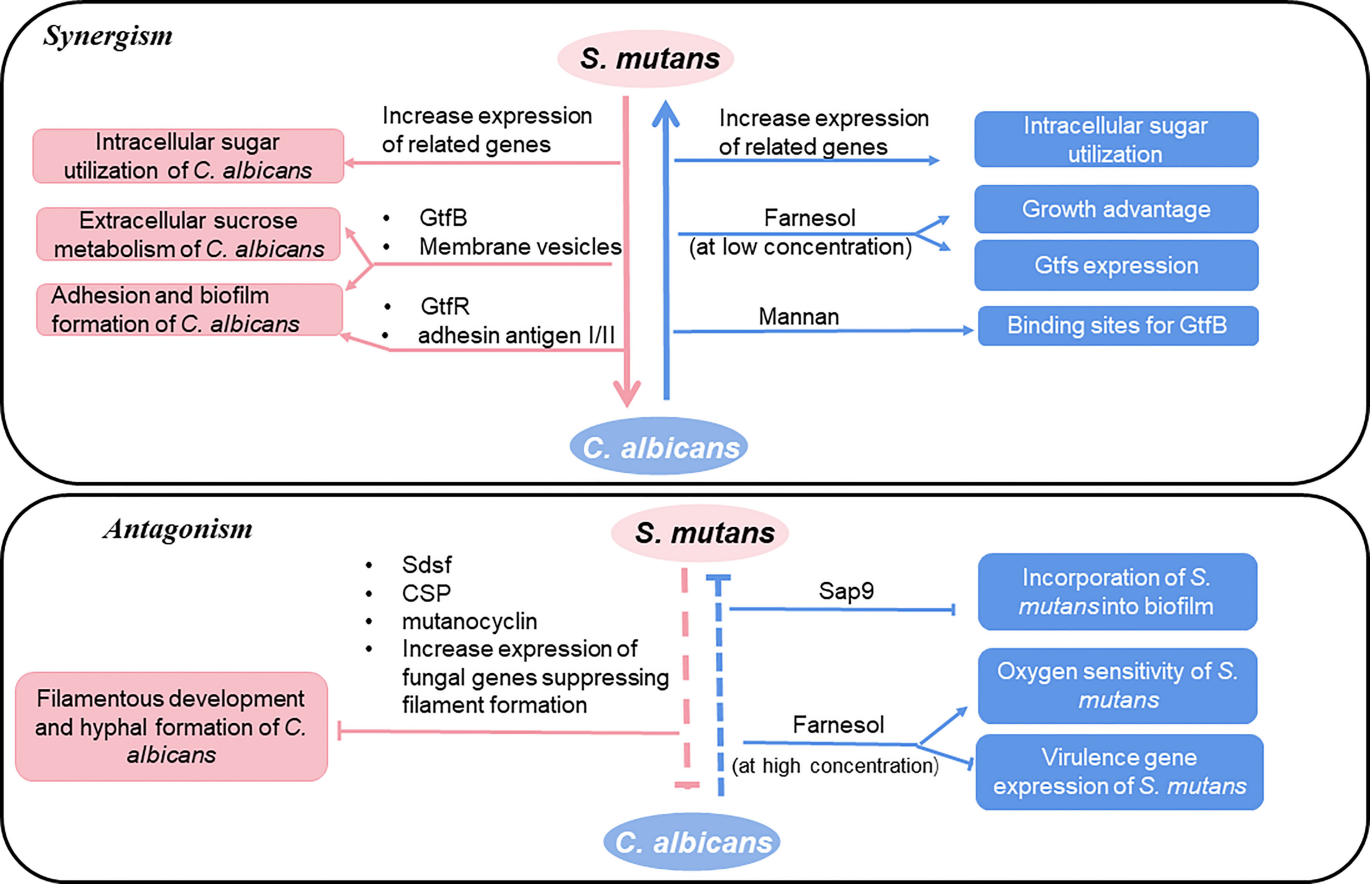
Interactions between *S. mutans* and *C. albicans*.

The copresence of *S. mutans* and *C. albicans* is frequently observed in oral samples from ECC patients (Xiao et al., 2018a). In recent years, the way how *S. mutans* and *C. albicans* interacts with each other as well as the effect of these interactions on the progress of dental caries including ECC has been intensively explored as summarized in **Figure 1**.

### 4.1 Synergism in metabolic activities

*C. albicans* promotes the carbohydrate intake of *S. mutans*. The glucose metabolic rate of *S. mutans* co-cultured with *C. albicans* was higher than those in the pure culture group (He et al., 2017; Oliveira et al., 2021). Further mechanism studies show that *C. albicans* affects the transcription of *S. mutans* genes associated with the transportation and metabolization of carbohydrates (He et al., 2017; Oliveira et al., 2021). Comparing to *C. albicans* or *S. mutans* single-species biofilms, dual species biofilms exhibited unique transcriptome profiles and most up-regulated genes were related to carbohydrate transport and metabolic/catabolic processes (Xiao et al., 2022). Specifically, when coexistence, many up-regulated genes of *S. mutans* are these participating in carbohydrate metabolism, galactose metabolism and glycolysis/gluconeogenesis (Xiao et al., 2022). Meanwhile, coculturing of *C. albicans* and *S. mutans* is a double win in terms of sugar utilization. Genes of *C. albicans* associating with carbohydrate metabolism were also significantly enhanced by co-culturing, including those involved in sugar transport, aerobic respiration, pyruvate breakdown, and the glyoxylate cycle (He et al., 2017; Oliveira et al., 2021). Moreover, a new cross-feeding mechanism between these two species were identified to be mediated by GtfB. GtfB secreted by *S. mutans* enhances *C. albicans* carbohydrate utilization (Ellepola et al., 2019). Membrane vesicles (MVs) are subcellular parts secreted by *S. mutans* cells. MVs containing the Gtf enzyme can locate in the extracellular matrix of *C. albicans* biofilm, which contributes to the sucrose metabolism of *C. albicans* (Wu et al., 2020).

### 4.2 Synergism through signal molecules

The quorum sensing molecule farnesol secreted by *C. albicans* is generally considered as a bacteriostatic substance (Fernandes et al., 2018). However, farnesol can promote the growth of *S. mutans*, the expression of *gtfs*, the formation of biofilm and biological colonies and the activity of transferrin (Kim et al., 2017). It is worth noting that the effect of farnesol on *S. mutans* is concentration-dependent. This molecule inhibits a series of life activities of *S. mutans* at a high concentration (> 100μM) (Kim et al., 2017). The specific action mechanism of farnesol on *S. mutans* is still unknown, and further study is needed.

### 4.3 Synergism during biofilm formation

The *C. albicans*-*S. mutans* dual biofilm is characterized by the interweaving of expanded *S. mutans* microcolonies with Candida yeasts, hyphae, and pseudo-hyphae to form a three-dimensional inter-kingdom superstructure (Negrini et al., 2022). Compared to *C. albicans* or *S. mutans* single species biofilm, dual-species biofilm shows increased biomass, viable cells, EPS and protein content, acid resistance, oxidation, and antibacterial stress resistance, and larger microcolonies as well as much more complex 3D structure (Falsetta et al., 2014; Lobo et al., 2019). The unique physical and chemical properties of *C. albicans*-*S. mutans* dual biofilm are resulted from the inter-kingdom synergistic interactions between these two microbes.

Polysaccharides are the major EPS components of biofilm playing important roles in the colonization of microorganisms. Polysaccharides produced by *S. mutans* are mainly β-1,3-glucan and β-1,6-glucan through the activity of Gtfs (Gregoire et al., 2011), and α-mannan is the most abundant EPS component of *C. albicans* biofilm, followed by β-1,6-glucan and β-1,3-glucan (Pierce et al., 2017). *S. mutans*-secreted Gtfs can bind strongly and stably to the mannan layer of *C. albicans*. This binding converts *C. albicans* into a de facto glucan producer and consequently promotes the assembly of the EPS-rich matrix scaffold. At present, it is known that three kinds of Gtfs can bind to the cell surface of *C. albicans in vitro*, among which GtfB has the strongest affinity. The binding of Gtfs to *C. albicans* affects the physical and chemical properties of dual biofilm significantly because, first, polysaccharides present in the biofilm directly affect the formation and size of the microcolony (Xiao et al., 2012). Second, the production of this 3D EPS-matrix contributes to form an intricate network of exopolysaccharide-enmeshed bacterial-islets (microcolonies) through localized cell-to-matrix interactions. Third, as a diffusion-limiting barrier, the EPS-matrix prevents acid within the biofilm from diffusing outward, thus prolonging and intensifying the acid attack. It helps to create spatial heterogeneities and acidic regions at specific locations at the surface of biofilm attachment (despite exposure to buffered neutral pH) (Xiao et al., 2017; Kim et al., 2018). Fourth, EPS-producing bacterial GtfB exoenzymes can directly modulate antifungal drug tolerance both at a single-cell level and within multicellular biofilms, even if *C. albicans* is defective in producing its own protective matrices (Liu et al., 2022). In addition, the binding of Gtfs to *C. albicans* enables the fungus to colonize EPS-coated surfaces readily, therefore recruiting more fungal cells into the biofilm. Finally, it enhances fungal-bacterial coherence (Falsetta et al., 2014). Consistent with this, mutants of *S. mutans* lacking glucosyltransferase gene showed the ability to seriously disrupt the colonization, accumulation, and formation of cospecies biofilms of *C. albicans* (Carvalho et al., 2006; Wan et al., 2021). *BCR1* is the key gene to develop the biofilm of *C. albicans*, whose major functional downstream targets include *HWP1*, *ALS1*, and *ALS3* genes that encode cell surface proteins. Interestingly, GtfB augments the *C. albicans* counterpart in mixed-species biofilms through a *BCR1*-independent mechanism (Ellepola et al., 2017). In addition, GtfR of *S. mutans* can provide adhesion conditions to increase the biomass of *C. albicans* and biofilm matrix (Souza et al., 2020). Membrane vesicles (MVs) are subcellular parts secreted by cells and are of significance in disease progression and intercellular communication. MVs containing the Gtf enzyme can locate in the extracellular matrix of *C. albicans* biofilm, which contributes to the sucrose metabolism of *C. albicans* (Wu et al., 2020). *S. mutans* can also mediate the recruitment of *C. albicans* into biofilm through its adhesin antigen I/II. *S. mutans* antigen I/II promotes the adhesion of *both S. mutans* and *C. albicans* (Yang et al., 2018). Antigen I/II mediated process is independent of the streptococcal receptors of *C. albicans* (such as Als1 and Als3 proteins).

## 5 Antagonism between *C. albicans* and *S. mutans*

Not only “peace” but also “war” exists between *C. albicans* and *S. mutans*. The *S. mutans*-*C. albicans* association has pleiotropic effects that could be both cooperative and antagonistic. *S. mutans* exerts an inhibitory effect on the morphogenesis, pathogenicity and biofilm formation of *C. albicans* (Vílchez et al., 2010; Barbosa et al., 2016). *S. mutans* inhibits the hyphal formation of *C. albicans* through streptococcus diffusible signal factor (Sdsf) and competence stimulating peptide (CSP). Sdsf is a fatty acid signaling molecule and an intermediate product of unsaturated fatty acid synthesis. It decreases the expression of *HWP1* and *SAP5* and therefore suppresses the transformation of *C. albicans* from yeast to hypha at a concentration that does not affect fungal growth (Lee et al., 2017). CSP not only inhibits the formation of germ tube but stimulates the transformation of mycelium to yeast (Satala et al., 2021). When mixed culturing, *S. mutans* also increases the expression of fungal genes which are known to suppress filament formation such as *tup1* and *nrg1* (Jarosz et al., 2009). CSP is a quorum sensing molecule produced by *S. mutans* at the beginning of the exponential growth stage. *S. mutans* affect the filamentous development of *C. albicans* also by secreting a secondary metabolite mutanocyclin (a tetra acid). It is well-known that the conserved camp/protein kinase A (PKA) pathway plays a central role in many life activities of *C. albicans* (Tao et al., 2022). Mutanocyclin can metabolize the subunit TPK2 in *C. albicans* in a dose-dependent manner by regulating the PKA pathway to inhibit the growth of filaments, and the inactivation of TPK2 also leads to an increase in the sensitivity of *C. albicans* to mutanomycin. Meanwhile, mutanocylin shows a global impact on the transcriptional profile of *C. albicans*, which mainly regulates cell wall components through the Ras1 camp/PKA signaling pathway and EFG1 as well as the subset of filamentous regulators regulated by SFL1. In addition, anchor protein genes related to cell wall biogenesis and remodeling may be involved in the regulation of mutanocyclin response, and anchor protein plays a key role in mutanocyclin regulated inflammation (Dutton et al., 2016).

*C. albicans* also has an inhibitory effect on *S. mutans*. For example, farnesol, the signaling molecule secreted by *C. albicans* at higher concentrations increases the oxygen sensitivity of *S. mutans* and down-regulated the expression of bacterial virulence-related genes including *luxS*, *brpA*, *ffh*, *recA*, *nth*, *smx*, *comC*, *comYB* as well as genes encoding bacteriocin. In addition, farnesol inhibits biofilm formation of biofilm (Rocha et al., 2018) and water-soluble EPS production (Wang et al., 2020). From this aspect, C. *albicans* suppress the cariogenic ability of *S. mutans* in specific situations. *C. albicans* that have the deletion of *Sap9* (encoding a secreted aspartyl protease) caused an increase in the incorporation of *S. mutans* into dual species biofilms, suggesting that *Sap9* may act as a key module to influence the competition between *C. albicans* and *S. mutans* (Dutton et al., 2016).

## 6 Summary and prospective

*S. mutans* has long been taken as the key etiological factor of dental caries, and the oral opportunistic fungus *C. albicans* also played an important role in promoting *S. mutans* to cause dental caries. At the same time, the studies related to ECC also reflect the correlation between them, but the specific interaction is unknown. Most of the released studies focus on gene regulation, adhesion, and metabolism interactions between these two microbes. With the innovation and development of technology, we need to explore the cariogenic mechanism and interaction of *S. mutans* and *C. albicans* in order to expand more ideas for the prevention and treatment of dental caries, formulate more detailed treatment strategies, and improve better oral health.

## Author contributions

YLu and YLin drafted the manuscript. ML and JH designed, edited, and added valuable insights to the manuscript. All authors contributed to the article and approved the submitted version.
